# 30580 A TL1 Approach to Assessing Peripheral Immune Changes in PTSD

**DOI:** 10.1017/cts.2021.628

**Published:** 2021-03-31

**Authors:** P. M. Mackie, C. Wilkinson, A. Gopinath, L. Knackstedt, H. Khoshbouei

## Abstract

ABSTRACT IMPACT: We present preliminary data and an outlined approach to assess peripheral immune changes associated with PTSD in a clinical setting and in a pre-clinical rat model of PTSD. OBJECTIVES/GOALS: We report our methodology and findings indicating a relationship between CNS dopamine signaling and peripheral immune cell populations and propose to extend this methodology to a PTSD patient population to elucidate immune-brain connections in this disorder. METHODS/STUDY POPULATION: Using an IRB-approved protocol in collaboration with a board-certified psychiatrist, we will recruit PTSD patients undergoing treatment, newly diagnosed drug-naiive PTSD patients, and age-matched healthy controls. Flow cytometry will be used for immunophenotyping on blood samples from each group. To complement this data, we will also measure serum cytokine levels in each group. In order to elucidate the connection between the observed immunophenotypes in the PTSD population and CNS neurotransmitters levels, we will employ a rodent model of PTSD and high-pressure liquid chromatography to measure dopamine levels in tandem with peripheral immune changes. RESULTS/ANTICIPATED RESULTS: In both humans and rodents with low CNS dopamine, an expansion of monocyte-derived suppressor cells was observed via flow cytometry. We anticipate that human PTSD patients will exhibit a similar expansion in suppressive immune cells’‘ in agreement with existing literature suggesting a chronic inflammatory state in PTSD. Moreover, in an animal model of PTSD we anticipate an inverse correlation between the CNS dopamine levels and the size of the immune suppressor cell population. DISCUSSION/SIGNIFICANCE OF FINDINGS: Our findings will indicate whether altered dopamine neurotransmission underlies peripheral immune system changes in the context of PTSD models and human patients. Thus, these findings will provide an alternative avenue for future investigations on the role of the immune system in PTSD.

